# Nodular Lymphoid Hyperplasia as Incidental Finding of Suspect Pulmonary Mass

**DOI:** 10.1155/2022/2242418

**Published:** 2022-05-14

**Authors:** Hannes Reuter, Stefan Reuter

**Affiliations:** ^1^Department III of Internal Medicine, University of Cologne, Kerpener Str. 62, Cologne 50937, Germany; ^2^Department of Internal Medicine 4, Klinikum Leverkusen, Am Gesundheitspark 11, Leverkusen 51375, Germany

## Abstract

Nodular lymphoid hyperplasia of the lung is a rare disease of polyclonal lymphoid proliferation. The incidental finding of a solid nodular lesion with irregular margins adjacent to the visceral pleura in the reported case was highly suggestive of malignancy. The present report underscores the typical immunohistochemical findings and the benign course of nodular lymphoid hyperplasia. The current knowledge about disease aetiology and the value of different diagnostic tools to distinguish nodular lymphoid hyperplasia from other pulmonary lymphoid lesions are summarized by a review of the literature. Surgical resection is not only diagnostic but also curative with no evidence so far that NLH can regress without operation. The present case shows the spontaneous regression of NLH after CT-guided biopsy indicating that an alternative, less invasive diagnostic approach has curative potential.

## 1. Introduction

Low-grade lymphoid proliferations in the lung, including those with reactive germinal centers, were commonly classified as low-grade B-cell lymphomas of mucosa-associated lymphoid tissue (MALT) [1]. However, Kradin and Mark were first to distinguish a small but distinct subset of lymphoid mass lesions that corresponded histologically to nodular hyperplasia of bronchus-associated lymphoid tissue (BALT) [2]. The unique entity and benign nature of this disorder has subsequently also been recognized by the World Health Organization (WHO), which introduced the term nodular lymphoid hyperplasia (NLH) of the lung [3].

## 2. Case Presentation

**Figure 1:**
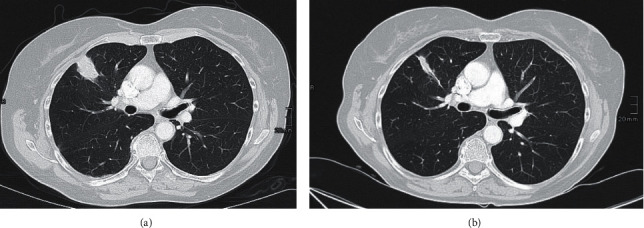
Chest computed tomography scans. (a) The initial scan shows a solid nodular lesion with irregular margins and adjacent to the visceral pleura in the right lower part of the upper lobe. (b) Five years later, the CT scan revealed only a small scar in the former lesion.

**Figure 2:**
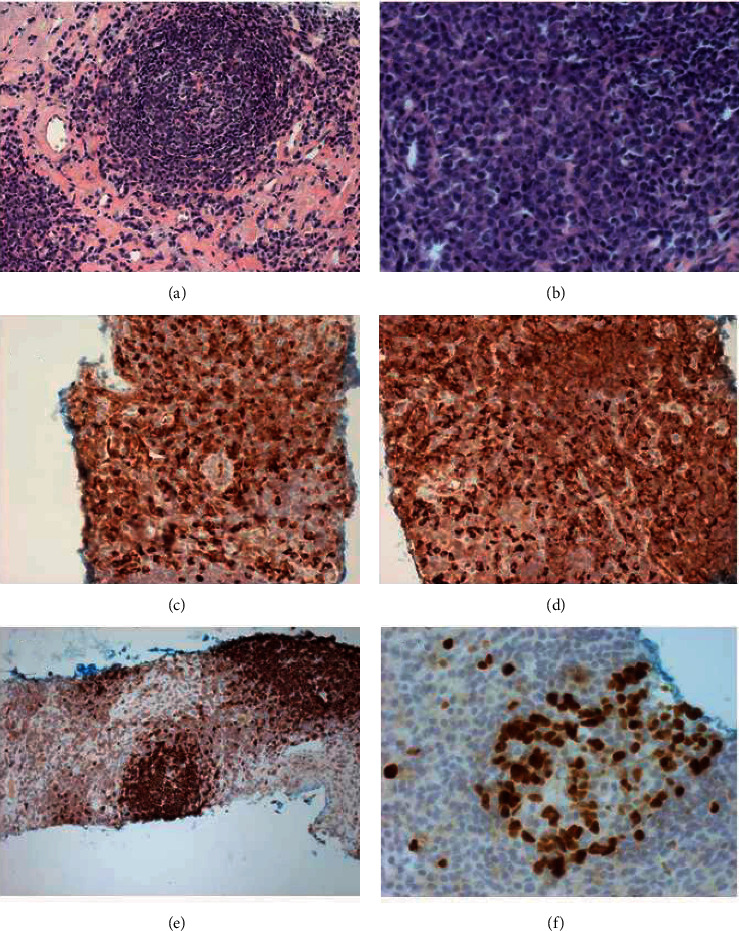
Histopathological appearances of the tumour. (a, b) Lymphoproliferative lesion was composed of dense infiltrates of mature lymphocytes and sporadic plasma cells along with lymphoid follicles with germinal centers. (a) Hematoxylin-eosin (H&E), ×10. (b) H&E, ×40. (c, d):Immunohistochemical (IHC) staining for immunoglobulin (Ig) *ĸ* (c) and Ig *λ*. (d) Light chains revealed polyclonal reactivity (×40). (e) IHC staining using CD20 antibodies showed high densities of B cells in reactive follicles. (f) Ki-67 IHC staining revealed high proliferative activity in the germinal center with no evidence of malignancy.

A 64-year-old female presented to the hospital because of suspected traumatic rib injury. She never complained a cough, no fever, weight loss, or night sweats. Chest X-ray did not reveal a fracture but unexpectedly showed a mass in the right upper lobe. This finding was confirmed by computed tomography (CT) showing a solid nodular lesion with irregular margins adjacent to the visceral pleura with a diameter of 1.2 inches in the upper right lobe (Figure 1). Under suspicion of malignancy, a CT-navigated core biopsy (diameter: 0.75 inches) was obtained showing well-defined lymphoid tissue masses with numerous reactive germinal centers, interfollicular lymphocytes, and plasma cells (Figure 2). Immunohistochemically, plasma cells that were reactive for both *κ*- and *λ*-light chain immunoglobulins supporting a polyclonal population (Figure 2(c) and 2(d)), and lymphocyte subset markers such as CD20 and CD10 identified B cells, which were negative for Bcl-2 (Figure 2(e)). CD3-positive T cells showed reactive patterns in the interfollicular zone. Ki-67 staining confirmed a high proliferative activity in reactive follicles with no evidence of malignancy (Figure 2(f)). These histological findings could exclude a lymphoma and resulted in the diagnosis of NLH. The postinterventional course was uneventful. A CT of the chest 5 years later confirmed the benign nature of the tumour with complete regression leaving only a small scar in the area of former lesion.

## 3. Discussion

The present case underscores the typical immunohistochemical findings and the benign course of NLH, a rare pulmonary disease with morphological features which are highly suggestive of malignancy. The disease was first described by Kradin and Mark in 1983 and is characterized by one or more benign nodules or localized lung infiltrates composed of reactive lymphoid cells [2].

In these mostly asymptomatic patients, the nodules are typically incidental findings in subpleural, occasionally peribronchial location [4]. Large airways involvement is uncommon. If symptoms occur they are unspecific, including cough, dyspnea, and pleuritic pain. Females are slightly more often affected than males in a ratio of 4 : 3. The age differs largely from 19 to 80 years (median 60 years). Mediastinal or hilar adenopathy is present in approximately one-third of patients [4]. The pathogenesis of NLH is unknown. Song et al. identified NLH in a patient with Sjogren's syndrome [5] although others did not suggest an association with collagen vascular disease [4].

The lesion is usually detected first in an X-ray or CT from the chest. However, there is no radiographic sign specific for NLH, especially in the distinction to malignant tumours. Based on a series of 67 patients, Fang et al. described the typical radiological manifestations of pulmonary NLH as solitary, or multinodular, solid or subsolid nodules with a wide array of additional radiographic findings, including lobulation, spiculation, vessel convergence, and pleural indentation as well as mediastinal or hilar lymph node involvement [6]. Even in 18F-fluorodeoxyglucose positron emission tomography (FDG-PET), the imaging findings can be very similar to those of lung cancer with varying FDG uptakes [7, 8]. Due to these radiographic signs of malignancy, most patients with NLH primarily undergo surgical lobectomy or sublobular resection of the lung without recurrence [4, 5].

To date, there is only little evidence of spontaneous regression of pulmonary NLH without operation. Some studies reported the regression of remaining lesions following the surgical resection of one nodule [9, 10] and another report described the reduction of an abnormal lung shadow in NLH induced by antibiotic treatment [11]. Surgical resection of NLH is therefore widely accepted not only as a diagnostic but also as a curative measure [6]. However, these patients would experience substantial loss of normal lung parenchyma for a benign condition, especially after complete lobectomy. To our knowledge, the present case is the first description of spontaneous regression of pulmonary NLH after CT-guided needle biopsy supporting an alternative, less invasive, and debilitating diagnostic approach with curative potential.

The histological picture of NLH is characterized by a dense nodular infiltration of mature, polyclonal lymphocytes and plasma cells with multiple reactive germinal centers, sharply demarcated from surrounding parenchyma and with central areas of scarring. Immunohistochemical staining shows a mixture of B cells with polytypic *κ*- and *λ*-light chain expression and T cells in the lymphoid infiltrate [4]. These histopathologic features need to be identified in order to distinguish NLH from other neoplastic lymphoproliferative pulmonary lesions. Differential diagnoses include the extranodal MALT lymphoma, which is similarly characterized by a mixed population of lymphoid cells with abundant plasma cells [12–14]. However, while NLH is a polyclonal lymphatic disorder, the MALT lymphoma shows monoclonal tumour cells [4, 15]. In addition, Dutcher bodies and pleural and bronchus invasion are common features of MALT lymphoma. Other low-grade lymphomas such as small lymphocytic lymphoma or chronic lymphocytic leukaemia could mimic NLH as well, but most such cases show a more diffuse infiltrative pattern rather than formation of well-defined nodules. Further differential diagnoses to be considered include the benign/non-neoplastic pulmonary lymphoid disorders such as lymphocytic interstitial pneumonia (LIP), follicular bronchiolitis (FB), or inflammatory pseudotumour [16–18]. LIP usually shows dense infiltration of plasma cells, lymphocytes, and histiocytes resulting in a diffuse alveolar widening [19]. In addition, the growth pattern is diffuse usually involving the entire lung, rather than nodular in appearance. FB is a lymphoid follicular hyperplasia with germinal centers as well, but can be distinguished from NLH by its location: while NLH is usually located in the subpleural area, and FB is typically distributed along the bronchiolar walls [19]. Some cases may exhibit overlapping features, and the distinction among these entities can be arbitrary. In these cases, the synopsis of clinical, radiographic, and histologic features may help to distinguish between entities.

## 4. Conclusion

NLH is a benign pulmonary disease with morphological features which are highly suggestive of malignancy. To date, the diagnosis is based on the typical histopathological findings with dense nodular infiltration of mature, polyclonal lymphocytes and plasma cells. Surgical resection of the tumour is the standard diagnostic and therapeutic approach to date, and there was no evidence so far that NLH can regress without operation. The present case shows the spontaneous complete reduction of NLH after CT-guided biopsy highlighting an alternative, less-invasive diagnostic approach with curative potential.

## Data Availability

The data are available on request by contacting the corresponding author.
